# Analysis of the curative effect of cervical spondylotic radiculopathy with osseous foraminal stenosis using ultrasonic osteotome in anterior cervical surgery

**DOI:** 10.1186/s12891-022-06083-1

**Published:** 2023-01-31

**Authors:** Junlin Liu, Qingquan Kong, Pin Feng, Bin Zhang, Junsong Ma, Yuan Hu

**Affiliations:** 1 Orthopaedic department, Hospital of Chengdu office of People’s Government of Tibetan Autonomous Region, Chengdu, 610041 Sichuan China; 2 Orthopaedic department, West China Hospital, Sichuan University, Chengdu, 610041 Sichuan China

**Keywords:** Anterior cervical surgery, Ultrasonic osteotome, Clinical efficacy, Surgical techniques

## Abstract

**Purpose:**

To explore the clinical efficacy and operation points of cervical radiculopathy with osseous foraminal stenosis treated with ultrasonic osteotome in anterior cervical surgery.

**Methods:**

From January 2018 to June 2021，a retrospective analysis of 23 patients with cervical radiculopathy with bony foraminal stenosis during this period was retrospectively analyzed. Anterior Cervical Discectomy and Fusion (ACDF) was used for all cases in this group. Intraoperative use of ultrasonic osteotome to decompress the nerve in the intervertebral foramina. The operation time, intraoperative blood loss and complication rate were recorded in this group of patients. Interbody fusion was evaluated using Brantigan criteria. The IC-PACS imaging system was used to measure the intervertebral foramen area (IFA) before and after surgery to evaluate the range of decompression. The VAS (Visual Analogue Scale, VAS) score and NDI (Neck Disability Index, NDI) score before and after surgery were recorded to evaluate the clinical efficacy.

**Results:**

All enrolled patients were followed up regularly for 1 year or more. The mean operative time was 61.5 ± 8.0 minutes. The average intraoperative blood loss was 88.3 ± 12.8 ml, and the average hospital stay was 8.1 ± 1.7d. Twenty one cases of successful fusion were followed up 1 year after operation, and the fusion rate was 91.3%. IFA expanded from 25.1 ± 4.0 mm2 before operation to 57.9 ± 3.4 mm2 at 1 year after operation, and the difference was statistically significant (*P* < 0.001). The VAS score and NDI score of patients 3 days after surgery, 3 months after surgery, and 1 year after surgery were significantly lower than those before surgery (*P* < 0.001). There was 1 case of dysphagia and 1 case of Cage subsidence after operation, and the complication rate was 8.6%.

**Conclusion:**

Anterior cervical surgery using ultrasonic osteotome in the treatment of cervical radiculopathy with bony foraminal stenosis has reliable clinical efficacy and high safety, and is worthy of clinical promotion.

## Introduction

Cervical radiculopathy shows the highest incidence rate amongst all forms of cervical spondylosis, reaching up to 60–70% [1]. Corresponding clinical symptoms occur due to the stenosis of the intervertebral foramen and compression of nerves. Due to rigid pressure factors, surgical decompression is often required to achieve a good clinical outcome. Anterior cervical discectomy and fusion (ACDF) is a classic surgical procedure to treat cervical spondylosis. It can directly relieve the compression of the anterior spinal cord, and its advantages over posterior surgery include less trauma and less bleeding [2–4]. In dealing with cervical radiculopathy accompanied by bony foraminal stenosis, the current methods of decompressing the intervertebral foramen through ACDF can be divided into indirect decompression and direct decompression. Indirect decompression mainly refers to the use of a larger cage to increase the height of the intervertebral space and increase the area of ​​the intervertebral foramen. However, osseous foraminal stenosis is often accompanied by osteophyte hyperplasia of the uncinate joint, so simply increasing the upper and lower diameters of the intervertebral foramen cannot achieve sufficient results [5]. The direct decompression method refers to the direct resection of the osteophyte ossification in the intervertebral foramen area. Currently, the decompression tools used clinically are mainly high-speed drills and spatulas. Be that as it is, when the bony stenosis in the intervertebral foramina is severe, the high-speed grinding drill is prone to complications such as Dural tear and nerve root injury due to its rolling and scraping effect which may cause the surgeon may not be able to perform adequate decompression due to unclear vision [6, 7]. Ultrasonic osteotome technology has been maturing in recent years, and various literature has reported that it can achieve good clinical results in posterior cervical laminoplasty [8–10]. Four years ago, our team tried to use ultrasonic osteotome to treat cervical radiculopathy with bony foraminal stenosis and accumulated some application experience. Therefore, we retrospectively analyzed this group of patients discussing its clinical efficacy and key points of operation.

## Materials and method

### Patients

This study is a retrospective study. All 23 patients enrolled underwent single-segment ACDF surgery in our hospital between January 2018 to June 2021, intraoperative ultrasound osteotome being used to reduce the foraminal area pressure. The operation time, intraoperative blood loss, incidence and complications were recorded. The Brantigan standard was used to evaluate the interbody fusion [11]. Fusion was classified into grades 1–5, of which grades 4 and 5 were successful fusion, and grades 1, 2, and 3 were fusion failure. Level 1 refers to intervertebral height loss, bone graft absorption, pseudoarthrosis. Level 2 refers to obvious light transmission area can be seen in the fusion area. Level 3 refers to suspected bone nonunion, with a small amount of light transmission area in the fusion area. Level 4 refers to suspicious bone fusion, obvious bone bridge formation in fusion area, and no light transmission area in fusion area. Level 5 refers to strong fusion, dense bone in the fusion area, obvious bone bridge forming a penetrating fusion area, and no light transmission area in the fusion area. The IC-PACS imaging system was used to measure the pre- and post-operative IFA in the cervical oblique radiograph and to evaluate the range of decompression. The VAS score and NDI score before and after surgery were recorded to evaluate the clinical efficacy. All procedures were performed by a chief physician with more than 20 years of surgical experience, and all experimental data was collected by two designated spine surgeons.

Inclusion criteria: 1. A clear diagnosis of single-segment cervical radiculopathy; all patients had unilateral symptoms, and unilateral foraminal decompression was performed during the operation; 2. Imaging showed bony foraminal stenosis; 3. The lesion site is the lower cervical vertebra (C3-C7); 4. The ultrasonic osteotome is used for decompression during the operation; Exclusion criteria: 1. Revision surgery; 2. The number of decompression segments exceeds 1; 3. Regular follow-up was not planned; 4. Those with mental illness or mental disorder; 5. The presence of intracranial or peripheral neuropathy; 6. The presence of contraindications to surgery or no surgery.

### Surgical technique

The patient was placed on the operating bed in supine, with soft pillows on both shoulders, and the head and neck are stretched backward. The surgical segment was positioned and marked by fluoroscopy. Intraoperative operations were started after routine disinfection and draping. The skin, subcutaneous tissue and platysma were incised, and the platysma and deep cervical fascia were released. Separation of structures was done along the upper edge of the scapula hyoid muscle with cutting of the muscle being avoided. The scapula hyoid muscle is pulled to the inside with a retractor and in the case of lower cervical spine surgery, it needs to be pulled to the outside together with the sternocleidomastoid muscle. The specific method implemented according to the shape of the muscle during the operation。Long vascular clamps were used to lift the prevertebral fascia and muscle and perform blunt dissection to expose the anterior longitudinal ligament. Positioning was then done at targeted segment by exposing the bony surface of either the vertebral body lying inferiorly or superiorly to the segment. Following positioning, the anterior longitudinal ligament, and the annulus fibrosus of the intervertebral disc were incised with a sharp knife, and the intervertebral disc tissue was excised with instruments such as curette, nucleus pulposus forceps, and laminar rongeurs, and the posterior longitudinal ligament and bilateral uncinate joints were exposed. The medial border of the uncinate joint was excised using an ultrasonic osteotome and the bony stenosis of the foraminal area underwent undercutting decompression. After the excision was completed, the posterior longitudinal ligament was incised with a hook knife, and the posterior longitudinal ligament and the remaining bony structures on the upper and lower edges of the vertebral body are gradually removed with a laminar rongeur. On completion of decompression, a nerve hook was used to check whether the decompression was adequate, also observing whether there is a notch or depression in the dura mater along with determining whether the spinal cord and nerve roots are adequately decompressed. Intervertebral fusion adopts common cage or zero-notch fusion cage. Ordinary cages are fixed with a plate-screw internal fixation system. C-arm fluoroscopy was done to ensure positioning was satisfactory. The wound was washed with normal saline, drainage was placed, and suturing was done. All patients underwent intradermal suture. On the first postoperative day, the drainage tube was removed if the drainage fluid was less than 50 ml. If the event of cerebrospinal fluid leakage, the removal of the drainage tube was delayed assuring good incision healing. On the 2nd day after operation, the frontal and lateral oblique X-ray of the cervical spine and the three-dimensional reconstruction CT were reviewed, and the cervical support was used to assist in movement if the positioning of the implant was satisfactory. On the 4-5th day after the operation, the patient could be discharged from the hospital if there were no abnormalities in the incision. Three months post-surgery, the cervical collar was removed provided interbody fusion was good, and normal neck activities were resumed.

### Statistical analysis

SPSS 20.0 statistical software was used for data analysis, measurement data were expressed as mean ± standard deviation, and paired t-test was used to compare continuous data before and after surgery. The test level was taken as two-sided α = 0.05.

## Results

### General results

The total number of enrolled patients was 23, including 14 males and 9 females, with an average age of 52.6 ± 9.8 years (range: 39–77 years). All patients underwent single-segment ACDF surgery, including 3 cases of C3/4 and 4 cases of C4/5, C5/6 was 12 cases, C6/7 was 4 cases, the average operation time was 61.5 ± 8.0 min, the average intraoperative blood loss was 88.3 ± 12.8 ml, and the average hospital stay was 8.1 ± 1.7d. All patients were followed up for more than 1 year (Table 1).

**Table 1:** Summary of the baseline data

Characteristics	ACDF(***n*** = 23)
**Age (years)**	52.6 ± 9.8
**Sex M/F**	14/9
**Surgical location**	
C3/4	3
C4/5	4
C5/6	12
C6/7	4
**Operating time (min)**	61.5 ± 8.0
**Blood loss (ml)**	88.3 ± 12.8
**Hospital stay(d)**	8.1 ± 1.7
**Follow up(Y)**	> 1

ACDF indicates Anterior Cervical Discectomy and Fusion; n indicates the total number of patients

## Functional results

The VAS score at 1 day before surgery was 5.9 ± 1.1, and the VAS score at 3 days, 3 months, and 1 year after surgery was 1.3 ± 0.9, 0.7 ± 0.6, and 0.4 ± 0.5, respectively. The NDI score at 1 day before surgery was 41.2 ± 2.9 points, and the NDI scores at 3 days, 3 months, and 1 year after surgery were 8.0 ± 1.6, 3.1 ± 1.5, and 1.6 ± 1.0 points, respectively. The VAS score and NDI score at each follow-up time point after operation were significantly lower than those before operation (Table 2).

**Table 2:** Comparison of the IFA between pre and postoperative and Degree of integration

Characteristics	Pre-op	Post 1y-op	P
**IFA (mm**^**2**^**)**	25.1 ± 4.0	57.9 ± 3.4	< 0.001
**Fusion**			
**Level 1**		0	
**Level 2**		0	
**Level 3**		2	
**Level 4**		11	
**Level 5**		10	
**Rate**		91.3%	

IFA indicates Intervertebral foramen area

The area of the intervertebral foramen was measured by cervical oblique X-ray using the IC-PACS imaging system, and the degree of intervertebral fusion was evaluated according to Brantigan’s criteria, in which grade 4–5 represents fusion success and grade 1–3 represents fusion failure

## Radiographic results

The preoperative IFA was 25.1 ± 4.0 mm2, and the postoperative IFA was 57.9 ± 3.4 mm2. The postoperative IFA was significantly enlarged compared with the preoperative ones. Twenty one cases of successful fusion were followed up 1 year after operation, and the fusion rate was 91.3% (Table 3).

**Table 3:** Comparation of the VAS and NDI between pre and postoperative

Characteristics	Pre-op	Post 3d-op	Post 3 m-op	Post 1y-op
**VAS upper limb** **NDI**	5.9 ± 1.1^*^ 41.2 ± 2.9^#^	1.3 ± 0.9^&1^ 8.0 ± 1.6^%1^	0.7 ± 0.6^&2^ 3.1 ± 1.5^%2^	0.4 ± 0.5^&3^ 1.6 ± 1.0^%3^

VAS indicates Visual Analogue Scale; NDI indicates Neck Disabilitv Index;

pre-op indicates preoperative; post-op indicates postoperative

*P* < 0.001,t 22.719 if &1 is compared with *;

P < 0.001, t 22.214 if &2 is compared with *;

P < 0.001, t 22.047 if &3 is compared with *

P < 0.001, t 48.647 if %1 is compared with #;

P < 0.001, t 60.607 if %2 is compared with #;

P < 0.001, t 60.888 if %3 is compared with #;

## Complications

There was 1 case of dysphagia after operation which was relieved within 1 week after operation. There was 1 case of Cage subsidence after operation with intervertebral fusion already having been achieved at 1-year follow-up. The complication rate was 8.6%.

### Typical case (Fig. 1)

**Fig. 1:**
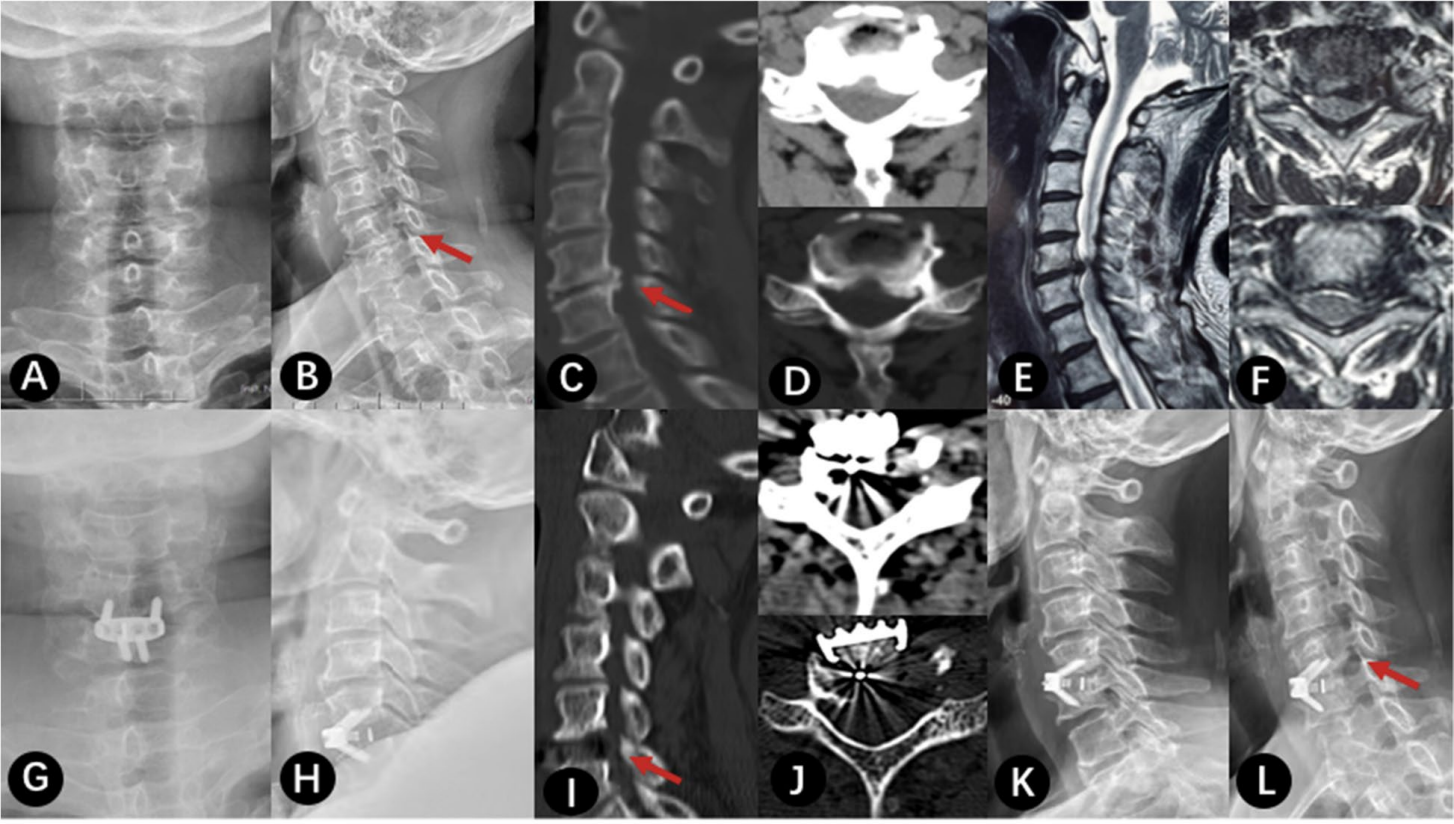
the patient, Female, 57 years old, was diagnosed with CSR(C5/6). **a**-**f**: Preoperative imaging studies. **a** and **b** showed cervical kyphosis, without rotation, slippage and scoliosis. **c** and **d** showed there are osteophytes at the posterior edge of the vertebral body, and the intervertebral foramen area is narrow. **e** and **f** showed the left intervertebral foramen area is obviously narrow and the C5 is compressed. **g**-**l**: were the postoperative imaging examination, in which **g**-**j** were the re-examination data 2 days after the operation, and **k**-**l** were the re-examination data 1 year after the operation. **i** and **j** showed the osteophyte at the posterior edge of the left vertebral body was completely removed, and the decompression range of the intervertebral foramen was satisfactory. **k**-**l** showed the intervertebral fusion was satisfactory, and the left intervertebral foramen area was significantly enlarged

## Discussion

Decompression of the intervertebral foramen is the focus of surgery for the treatment of cervical spondylosis of radiculopathy. However, when the uncinate joint osteophytes or the ossification of the posterior edge of the vertebral body lead to stenosis of the intervertebral foramen, the surgical difficulty will increase significantly. At present, there are many ways to decompress the intervertebral foraminal area, but they are all controversial. Koc uses the lateral-anterior approach to decompress the intervertebral foramen area. Although it has achieved good results [12], the exposed surgical field is small, and bilateral decompression cannot be performed simultaneously, so the range of surgical indications is small. In addition, the surgical target is close to the vertebral artery, and the risk of vascular injury is high. Compared with the anterior approach, the decompression of the intervertebral foramen area by the posterior approach is safer [13], but because the ventral bony compression cannot be completely removed, the effect is not satisfactory, and it is prone to recurrence with a second anterior approach surgery being required to achieve adequate decompression. In recent years, posterior cervical minimally invasive surgery is also commonly used to treat cervical radiculopathy, but its main technical point is to excise the prominent nucleus pulposus tissue, and its use value is low when accompanied by bone compression factors. At present, ACDF surgery is the most used surgical method for cervical radiculopathy with bony foraminal stenosis. However, during decompression, the foramen area is prone to bleeding and the visual field is blocked making the use of traditional grinding and scraping difficult. Decompression with a spoon is more difficult therefore osteophytes or ossification residues are prone to occur, and the risk of nerve damage is increased [14, 15].

**Fig. 2:**
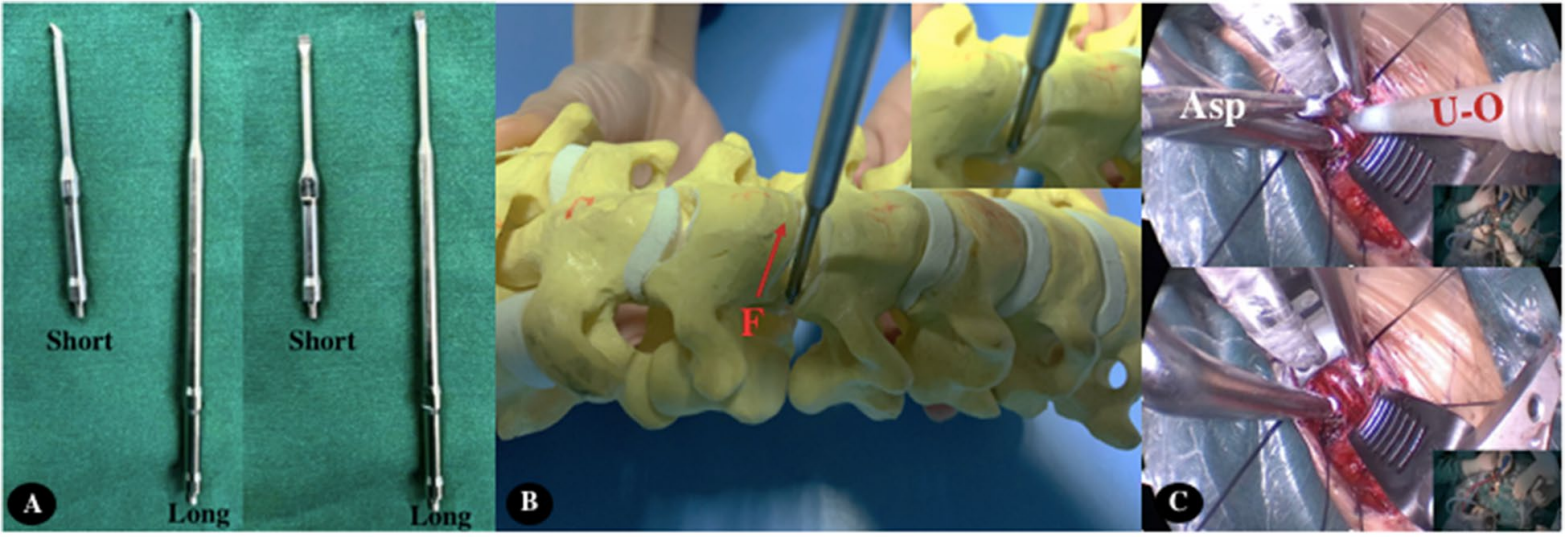
F stands for Force. A stands for Aspirator. U-O stands for ultrasonic osteotome. **a** represents Ultrasonic bone osteotome with spoon shaped tip. **b** is a model figure. The red arrow in the figure shows that the ultrasonic osteotome applies force to the dorsal side of the nerve. **c** shows the intraoperative image. The operator uses the ultrasonic osteotome with one hand, the auxiliary hand uses the aspirator, and both hands operate simultaneously to improve the surgical efficiency

Ultrasonic osteotome is a new type of osteotomy device that has emerged in recent years. Its safe use method effectively solves the shortcomings of traditional drills and curettes in decompressing the intervertebral foramen [16–18]. First, in cervical spondylotic radiculopathy, the hyperplastic osteophyte will squeeze the Batson venous plexus while compressing the spinal cord or nerve root to cause symptoms, thus affecting the return flow. Some patients can see the tension of the epidural vein during the operation, so the spinal canal decompression, especially the intervertebral foramen decompression process often touches the varicose venous plexus and causes bleeding [19, 20]. The direction of the force applied when using traditional instruments is parallel to the spinal cord or toward the spinal cord, because when the force toward the nerve direction is used, it is easy to cause grinding drill leakage and nerve damage. In addition, the use of high-speed grinding drill requires a high degree of clarity in the visual field, because when the visual field is blurred, it will be difficult for the operator to judge the positional relationship between the grinding head and the spinal cord，which increased the risk of nerve injury [21]. Curettes are applied in a direction parallel or dorsal to the spinal cord, which can effectively reduce the risk of nerve injury, but it is inefficient in removing bony compressions and cannot be used as a primary decompression tool. The ultrasonic osteotome with a spoon-shaped blade uses the force away from the spinal cord to remove osteophytes by a method similar to an electric curette. This method can completely separate the nerve and vascular tissue from the blade and can completely avoid neurovascular damage. Bone compressions can be safely removed even when the field of view is unclear. In addition, ultrasonic osteotome can reduce bleeding while cutting bone tissue. Its principle is ultrasonic cavitation effect [22, 23], which can vaporize water in tissues, break and denature protein hydrogen bonds, disintegrate cells, coagulate and denature hemoglobin, and play a hemostatic role. We found during the operation that when decompressing the intervertebral foramen area, the bleeding did decrease compared with traditional instruments, which is consistent with other literature reports [24]. In this group of trials, all patients had no symptoms of nerve injury, and the results also indicated that the ultrasonic osteotome was safe in decompressing the intervertebral foramen area. Secondly, the ultrasonic osteotome has the ability to decompress the intervertebral foramen. The bony pressure in the intervertebral foramen area is often not fully exposed. At this time, the grinding cannot be used to decompress the intervertebral foramen area because the depth cannot be confirmed, often leading to risk of vascular and nerve damage being significantly increased. Ultrasonic osteotome can use electric curette in undercutting decompression without fully revealing the bony compression. When the decompression range is completed according to the preoperative planning, the nerve hook can be used to check whether the decompression is sufficient to ensure the operation effect. In this group of trials, the VAS score and NDI score were significantly improved compared with those before surgery, which indicated that the use of ultrasonic osteotome to decompress the cervical spondylotic radiculopathy with bony foraminal stenosis in ACDF surgery is reliable. In addition, there was no residual bone compression material in this group of patients, and the size of the intervertebral foramen of 57.9 ± 3.4 mm2 after operation was significantly enlarged compared with that before operation, which proved that the decompression range was satisfactory from the perspective of imaging (Fig. 2).

Combined with the experience of intraoperative ultrasonic osteotome, we believe that the use of ultrasonic osteotome in the decompression of the intervertebral foramen area with bony stenosis has the following points. The first is to choose the correct cutter head. The long-handled spoon-shaped cutter head is the first choice. The spoon-shaped cutter head ensures the function of the ultrasonic bone knife similar to the electric curette, and the long handle can avoid the occlusion of the field of vision and facilitate the operation. The second is to ensure that the mid part of the spoon-shaped cutter head needs to contact the bone surface each time of curettage to avoid damage to the vertebral artery and nerve root adjacent to the intervertebral foramen area [14]. The third is to gradually expand the decompression. When the preoperative planning decompression range is almost reached, after each use of the ultrasonic osteotome to cure the bony oppression, it is necessary to use the nerve hook to determine whether the decompression is sufficient, to avoid excessive decompression leading to bone loss and vertebral artery damage. The fourth is to fully protect the ultrasonic bone cutter head. During the operation, we found that the cutter head is easily damaged if it comes into contact with metal substances, such as attractors and retractors. Therefore, it is necessary to avoid contact with metal substances during the operation. Our experience is that when decompressing the intervertebral foramen, the suction can be replaced with non-metallic materials to avoid damage. Fourth, local high temperature will be formed on the surface of the ultrasonic osteotome tip. Although the water spraying function of most ultrasonic osteotomes can reduce local temperature at present, staying at the same position for a long time may still burn nerve tissue [25]. We have not seen any similar cases in this trial, which we believe is due to our preventive measures for high-temperature scald during operation. First of all, we will try to reduce the decompression time of the same part to avoid excessive temperature. Secondly, local water shall be sucked away in time to give full play to its cooling effect. Finally, avoid direct contact between the back of the knife head and the nerve to avoid direct scalding [26].

## Conclusion

This study shows that the application of ultrasonic osteotome in anterior cervical surgery in the treatment of cervical radiculopathy with osseous foraminal stenosis is reliable and safe. It should be based on mastering the skills of using ultrasonic osteotome used in clinical practice.

## Data Availability

The datasets analysed in this article are available from the corresponding author on reasonable request.

## Abbreviations

ACDF: Anterior Cervical Discectomy and Fusion
IFA: Intervertebral foramen area
VAS: Visual Analogue Scale
NDI: Neck Disability Index
